# Editorial: Pediatric critical care in low resource settings

**DOI:** 10.3389/fped.2024.1420688

**Published:** 2024-07-08

**Authors:** J. C. J. Calis, R. A. Bem, M. J. Chisti

**Affiliations:** ^1^ Department PICU, Emma Childrens' Hospital of the Amsterdam UMC - location Meibergdreef, Amsterdam, Netherlands; ^2^ Department of Paediatrics and Child health, Kamuzu University of Health Sciences, Blantyre, Malawi; ^3^ Department of PICU, ICDDR, B, Dhaka, Bangladesh

**Keywords:** global health, pediatric critical care, low- and middle-income countries (LMIC), research, paediatric intensive care unit

**Editorial on the Research Topic** Pediatric critical care in low resource settings

Global paediatric mortality rates have shown a promising decline over the last three decades (1). During this period, paediatric mortality has halved, and this is largely attributed to advances in preventive healthcare. However, to further diminish these rates, attention must be directed towards enhancing in-hospital care. A pivotal aspect of this improvement lies in bolstering paediatric critical care services.

This trend towards prioritizing critical care was already evident before the onset of the COVID-19 pandemic, but the crisis starkly underscored its significance, particularly concerning adult care. Several low- and middle-income countries have formulated or significantly increased their critical care agendas, yet there is much still to be done (2). Although these agendas focus mostly on adults, it may be more ethical, relevant, and effective to focus on children as they are vulnerable, their mortality rates are high, and childhood illness costs the most disability-adjusted life years (DALYs) (3).

Upgrading paediatric critical care facilities in low- and middle-income countries presents multifaceted challenges. These include issues such as educational gaps in training personnel for this specialized field, logistical hurdles in ensuring the availability of new essential equipment and supplies, and complex infrastructure requirements, ranging from oxygen plants to equipment maintenance (2, 4, 5). Furthermore, the expansion of critical care services brings about transformative changes in clinical possibilities while also introducing new ethical dilemmas (6).

Despite these challenges, there are signs of progress in recent years. Paediatric intensive care units are beginning to emerge in Africa and see an increase in presence and growth in low- and middle-income countries (4, 5, 7). However, to effectively develop these critical care improvements, robust evidence-based practices are needed. Despite this, research in this area lags behind the expansion of paediatric critical care capacity (8, 9). In this research topic, we provided a platform to share knowledge concerning critical care in low- and middle-income settings.

This research topic presents articles that explore the similarities and differences in delivering paediatric critical care in low- and middle-income settings compared to high-income settings. In some paediatric critical units, clinical challenges and findings are similar to those in high-income settings: extubation failure in a paediatric intensive care unit (PICU) in Thailand, which showed comparable frequency to high-income settings Saengsin et al. The findings from a NICU in Ethiopia show that clinical problems, such as neonatal sepsis, are also important yet much more frequent as they report a 40% prevalence Mezgebu et al. The article about infectious encephalopathy in four African countries shows that similar clinical problems occur, yet have other aetiologies than in high-income settings Bacha et al. Finally, specific tropical diseases can be studied using (bedside) diagnostics, as shown in an article about optical nerve sheath diameter and cerebral malaria Raees et al. All in all, these diverse studies underline that research is essential to further paediatric critical care in low- and middle-income countries.
